# Route of Feeding as a Proxy for Dysphagia After Stroke and the Effect of Transdermal Glyceryl Trinitrate: Data from the Efficacy of Nitric Oxide in Stroke Randomised Controlled Trial

**DOI:** 10.1007/s12975-017-0548-0

**Published:** 2017-08-02

**Authors:** Lisa J Woodhouse, Polly Scutt, Shaheen Hamdy, David G Smithard, David L Cohen, Christine Roffe, Daniel Bereczki, Eivind Berge, Christopher F Bladin, Valeria Caso, Hanne K Christensen, Rónán Collins, Anna Czlonkowska, Asita de Silva, Anwar Etribi, Ann-Charlotte Laska, George Ntaios, Serefnur Ozturk, Stephen J Phillips, Kameshwar Prasad, Szabolcs Szatmari, Nikola Sprigg, Philip M Bath

**Affiliations:** 1 Stroke Trials Unit, Division of Clinical Neuroscience, University of Nottingham, City Hospital Campus, Hucknall Road, Nottingham, NG5 1PB UK; 2 Centre for Gastrointestinal Sciences, University of Manchester, Salford, UK; 3 Elderly Medicine, Princess Royal University Hospital, Orpington, UK; 4 Elderly Medicine, Northwick Park Hospital, Harrow, UK; 5 Institute for Science and Technology in Medicine, Keele University, Stoke-on-Trent, UK; 6 Neurology, Semmelweis University, Balassu, Budapest, Hungary; 7 Oslo University Hospital, Oslo, Norway; 8 The Florey Institute of Neuroscience & Mental Health Melbourne Brain Centre—Austin Campus, Heidelberg, Australia; 9 Stroke Unit, Ospedale Santa Maria della Misericordia di Perugia, Perugia, Italy; 10 Neurology, Bispebjerg Hospital & University of Copenhagen, Copenhagen, Denmark; 11 Stroke Service, Tallaght Hospital, Tallaght Dublin, Ireland; 12 Neurology 2, Institute of Psychiatry and Neurology, Warsaw, Poland; 13 Clinical Trials Unit, University of Kelaniya, Ragama, Sri Lanka; 14 Neurology, Ainshams University, Cairo, Egypt; 15 Clinical Sciences, Karolinska Institute, Stockholm, Sweden; 16 Medicine, University of Thessaly, Larissa, Greece; 17 Neurology, Selcuk University, Konya, Turkey; 18 Division of Neurology, Halifax Infirmary, Halifax, Canada; 19 Neurology, All India Institute of Medical Sciences, New Delhi, India; 20 Neurology, Clinical County Emergency Hospital, Targu Mures, Romania; 21 Stroke, Nottingham University Hospitals NHS Trust, Nottingham, UK

**Keywords:** Dysphagia, Glyceryl trinitrate, Outcome, Randomised controlled trial, Stroke

## Abstract

Post-stroke dysphagia is common, associated with poor outcome and often requires non-oral feeding/fluids. The relationship between route of feeding and outcome, as well as treatment with glyceryl trinitrate **(**GTN), was studied prospectively. The Efficacy of Nitric Oxide in Stroke (ENOS) trial assessed transdermal GTN (5 mg versus none for 7 days) in 4011 patients with acute stroke and high blood pressure. Feeding route (oral = normal or soft diet; non-oral = nasogastric tube, percutaneous endoscopic gastrostomy tube, parenteral fluids, no fluids) was assessed at baseline and day 7. The primary outcome was the modified Rankin Scale (mRS) measured at day 90. At baseline, 1331 (33.2%) patients had non-oral feeding, were older, had more severe stroke and more were female, than 2680 (66.8%) patients with oral feeding. By day 7, 756 patients had improved from non-oral to oral feeding, and 119 had deteriorated. Non-oral feeding at baseline was associated with more impairment at day 7 (Scandinavian Stroke Scale 29.0 versus 43.7; 2*p* < 0.001), and worse mRS (4.0 versus 2.7; 2*p* < 0.001) and death (23.6 versus 6.8%; 2*p* = 0.014) at day 90. Although GTN did not modify route of feeding overall, randomisation ≤6 h of stroke was associated with a move to more oral feeding at day 7 (odds ratio = 0.61, 95% confidence intervals 0.38, 0.98; 2p = 0.040). As a proxy for dysphagia, non-oral feeding is present in 33% of patients with acute stroke and associated with more impairment, dependency and death. GTN moved feeding route towards oral intake if given very early after stroke.

**Clinical Trial Registration**

Clinical Trial Registration-URL: http://www.controlled-trials.com. Unique identifier: ISRCTN99414122.

**Electronic supplementary material:**

The online version of this article (doi:10.1007/s12975-017-0548-0) contains supplementary material, which is available to authorized users.

## Introduction

Dysphagia (difficulty in swallowing) is a common complication after stroke affecting up to 65% of patients, many of whom are asymptomatic [1] or have symptoms that are not thought to be related to swallowing problems. Although many patients recover swallowing spontaneously, 11–50% still have dysphagia at 6 months [2, 3]; conversely, a significant proportion of patients are able to swallow safely within 1–2 weeks [1, 3]. Post-stroke dysphagia (PSD) is associated with a poor outcome for multiple reasons: first, it is a manifestation of severe stroke and therefore is associated with increased death, dependency, disability, impairment and institutionalisation; [4] second, it causes aspiration of foods, liquids and oral secretions and therefore pneumonia [5–7], which in itself leads to death; [6] and third, poor recognition and management leads to dehydration and malnutrition. In the acute phase, the presence of dysphagia leads to changes in the feeding of patients from oral routes to the use of enteral feeding tubes or parenteral fluids.

Although multiple advances have been made in the very early management of stroke (e.g. with thrombolysis, aspirin, mechanical thrombectomy and hemicraniectomy) and secondary prevention (antithrombotics, blood pressure lowering, lipid lowering, carotid endarterectomy), PSD remains a neglected research area and its optimal management, including treatment, have yet to be defined. Nevertheless, stroke guidelines recommend assessment of swallowing within 24 h and patients with an unsafe swallow are recommended to be nil by mouth; these recommendations constitute a key performance indicator in many stroke services. A number of trials have investigated the treatment of dysphagia, including acupuncture, behavioural therapy, physical stimulation, neuromuscular electrical stimulation and pharyngeal electrical stimulation [8–11], and several have given encouraging results [12]. In respect of drug treatment, a small pilot randomised trial suggested that nifedipine (a calcium channel blocker that relaxes oesophageal smooth muscle) might improve swallowing, and metoclopramide (a dopamine D2-receptor antagonist with antiemetic and gastric prokinetic activity) might reduce the incidence of pneumonia [13, 14]. Conversely, lisinopril (an angiotensin converting enzyme inhibitor) failed to prevent pneumonia [15].

Here we describe the natural history and outcomes of patients with feeding problems in the acute phase of stroke using data from the large ‘Efficacy of Nitric Oxide in Stroke’ (ENOS) trial [16–19]. We also report the effect of transdermal glyceryl trinitrate (GTN, a nitric oxide (NO) donor that relaxes smooth muscle) hypothesising that it might improve the oesophageal phase of swallowing and therefore return patients to oral feeding and a normal diet [17]. Of relevance, loss of a1ß1 soluble guanylate cyclase, the major NO receptor, leads to moyamoya and achalasia [20], the latter demonstrating the potential relationship between NO and swallowing. Since GTN might improve functional outcome if administered very early (<6 h), as seen in the ENOS and RIGHT trials [19, 21, 22], we have also assessed its effect on feeding route in this subgroup of patients.

## Methods

### ENOS Trial

The protocol, statistical analysis plan, baseline characteristics and main results for ENOS (ISRCTN99414122) have been published previously [16–19]. Brief information on the trial design is given in the Supplement.

### Route of Feeding and Definitions

ENOS did not record specific information on dysphagia or aspiration but collected data on route of feeding at baseline and day 7 [17]. As such, feeding route is a clinical consequence of dysphagia and its clinical recognition. Feeding route was defined using a pragmatic six-level ordered categorical scale comprising normal diet, soft diet, nasogastric tube, percutaneous endoscopic gastrostomy tube, intravenous or subcutaneous fluids and no feeding/fluids [17]. At day 7, death was added as a seventh level. This scale has not been used before or validated in the context of clinometric aspects such as face, content and construct validity. The explanation for no feeding or fluids was not collected but may reflect treatment for cerebral oedema or palliation.

Binary analyses were performed on oral feeding (normal and soft diet) versus non-oral feeding/fluids (nil by mouth = enteral tube, parenteral fluids, no feeding/fluids and death if at day 7), and this binary information was used as a minimisation variable at the time of randomisation [17, 19]. Definitions for other outcomes are given in the statistical analysis plan [17]. Information on pneumonia and chest infection was obtained from serious adverse event (SAE) reports as determined by local investigators and no formal definitions of these events were set; SAEs were adjudicated by experts blinded to treatment allocation. Respiratory tract infection (RTI) was considered as a composite of pneumonia, chest infection and/or exacerbation of chronic obstructive pulmonary disease (COPD).

### Statistics

All analyses were exploratory, by intention to treat, and no correction for multiplicity of testing was made. Data are shown as number (%) or mean (standard deviation). Comparisons between groups used binary logistic regression, Cox proportional hazards regression (death), ordinal logistic regression (OLR, for ordered categorical variables, e.g. feeding route) or multiple linear regression (continuous or pseudo continuous data such as modified Rankin Scale [mRS], Barthel Index [BI], health utility status). Results are given as odds ratio (OR) or mean difference (MD), with 95% confidence intervals (95% CI); *p* < 0.05 is considered significant. The assumption of proportionality of odds for OLR was tested using the likelihood ratio. Analyses were performed using SAS version 9.3.

## Results

### Feeding Route

Since feeding route was a minimisation variable used at the time of randomisation, baseline information was available for all 4011 patients enrolled into ENOS. 43.3% of patients were on a normal diet at baseline, with 23.5% on a soft diet, 5.6% receiving fluids and/or food via a nasogastric tube, 0.2% food and/or fluids by a gastrostomy feeding tube, 18.1% receiving parenteral fluids and 9.3% no fluids or food by any route (Table 1). When aggregating feeding route into patients receiving oral (normal or soft diet) versus non-oral nutrition/fluids (tube feeding, parenteral fluids or none), one third of patients were not taking fluids or food by mouth; just 43.3% of patients were on a normal diet at baseline with 56.7% of patients exhibiting some form of swallowing abnormality; and altogether, 66.8% of patients were taking some form of oral feeding, and 33.2% no oral feeding (Supplementary Fig. I). Patients on non-oral feeding were significantly older (+5.4 years), more likely to be female (+6.9%) and have a history of hypertension (+3.9%) and have more severe stroke (SSS −13.7 points, TACS +36.3%) and high systolic blood pressure and heart rate (+2.4 mmHg, +1.8 beats per minute) on inclusion to the study (Table 1). Similar findings were seen in patients randomised within 6 h of onset (Supplementary Table I).

**Fig. 1:**
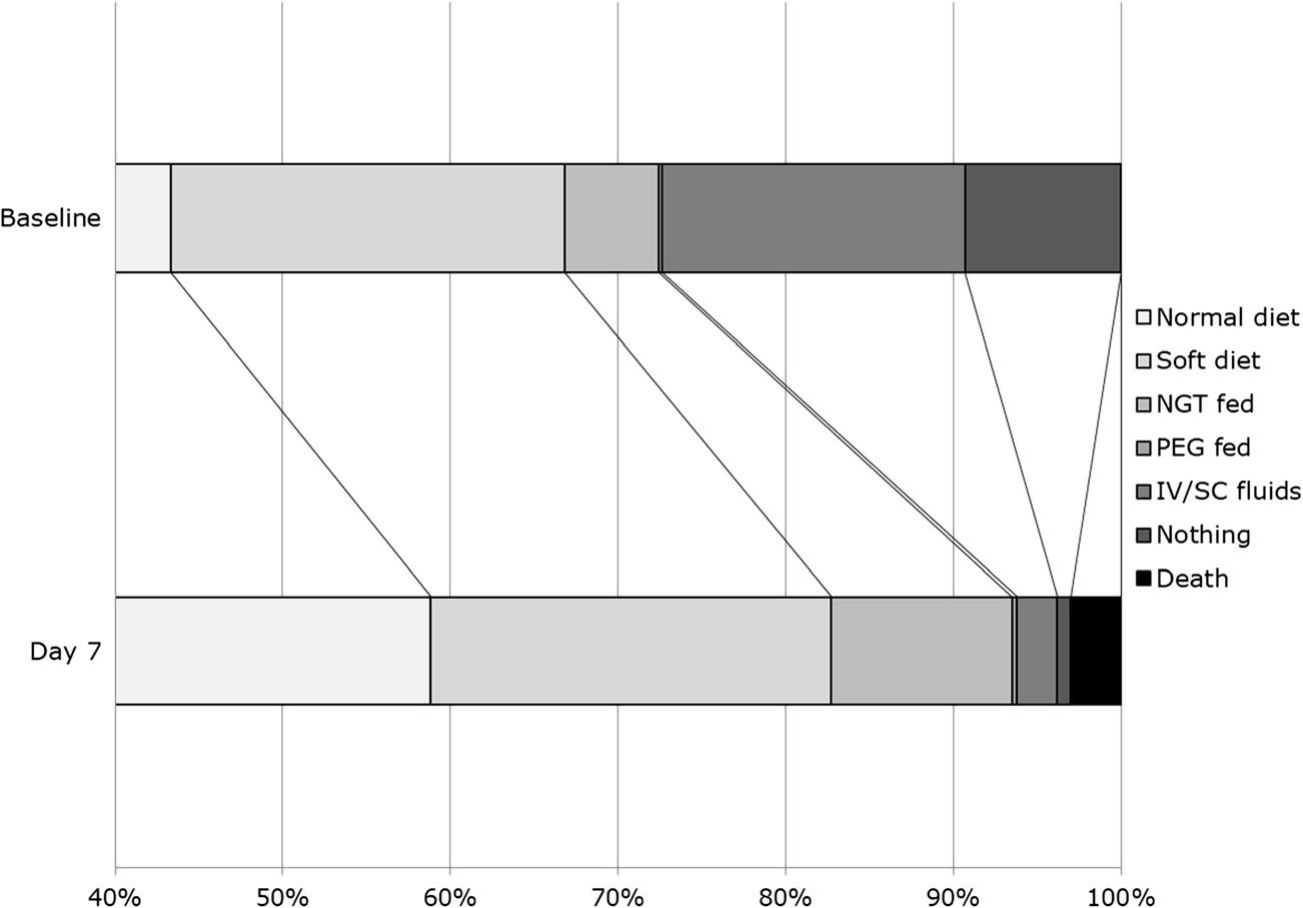
Change in feeding route from baseline to day 7 for all patients. Comparison by adjusted ordinal logistic regression. Note: the first 40% of patients were all on normal diet and are not shown for clarity. Adjusted common odds ratio 2.67, 95% CI 2.44–2.93, 2*p* < 0.001

Data on feeding route at day 7 were missing for 14 (0.35%) patients. By day 7, 756 of 1328 (56.9%) patients had improved from non-oral to oral feeding whereas 119 of 2669 (4.5%) had deteriorated moving from oral to non-oral feeding, or had died (Supplementary Table II). Overall, there was a significant move to improved feeding route over the first 7 days of monitoring, adjusted common odds ratio 2.67, 95% CI 2.44–2.93, 2p < 0.001 (Fig. 1). Data on feeding route beyond day 7 were not collected.

**Table 1:** Baseline characteristics of patients on oral versus-non oral-feeding/fluids at baseline

	All	Non-oral	Oral	Difference (95% CI)	2*p*
Number of participants	4011	1331	2680		
Age (years)	70.3 (12.2)	73.9 (11.6)	68.5 (12.1)	5.4 (4.6, 6.2)	<0.001
Sex, male (%)	2297 (57.3)	701 (52.7)	1596 (59.6)	−6.9 (−10.1, −3.6)	<0.001
Medical history (%)					
Previous stroke	594 (14.8)	192 (14.4)	402 (15.0)	−0.6 (−2.9, 1.7)	0.63
Hypertension	2607 (65.0)	900 (67.6)	1707 (63.7)	3.9 (0.8, 7.0)	0.014
Ischaemic heart disease	669 (16.7)	228 (17.1)	441 (16.5)	0.8 (−1.8, 3.3)	0.55
Scandinavian Stroke Scale	33.7 (13.2)	24.6 (12.4)	38.3 (11.0)	−13.7 (−14.5, −12.9)	<0.001
Blood pressure (mmHg)					
Systolic	167.2 (19.0)	168.8 (19.4)	166.5 (18.7)	2.4 (1.1, 3.7)	<0.001
Diastolic	89.5 (13.1)	89.0 (13.3)	89.8 (13.0)	−0.8 (−1.7, 0.1)	0.069
Heart rate (bpm)	77.5 (14.7)	78.7 (15.8)	76.9 (14.1)	1.8 (0.8, 2.8)	<0.001
Stroke type (%)					
Ischaemic	3342 (83.3)	1086 (81.6)	2256 (84.2)	−2.6 (−5.1, −0.1)	0.039
Haemorrhagic	629 (15.7)	234 (17.6)	395 (14.7)	2.8 (0.4, 5.3)	0.020
Time to randomisation (hours)	26.0 (12.9)	25.4 (12.9)	26.3 (12.8)	−0.8 (−1.7, 0.0)	0.052
Syndrome (%) [23]					
Total anterior	1209 (30.1)	724 (54.4)	485 (18.1)	36.3 (33.3, 39.3)	<0.001
Partial anterior	1251 (31.2)	378 (28.4)	873 (32.6)	−4.2 (−7.2, −1.2)	0.007
Posterior	154 (3.8)	25 (1.9)	129 (4.8)	−2.9 (−4.0, −1.8)	<0.001
Lacunar	1397 (34.8)	204 (15.3)	1193 (44.5)	−29.2 (−31.9, −26.5)	<0.001
Symptoms (%)					
Dysphasia	1610 (40.1)	813 (61.1)	797 (29.7)	31.3 (28.2, 34.5)	<0.001
Neglect	1068 (29.9)	601 (55.1)	467 (18.8)	36.3 (33.0, 39.6)	<0.001
Side of lesion (%)					
Right	2091 (52.1)	645 (48.5)	1446 (54.0)	−5.5 (−8.8, −2.2)	0.001
Left	1903 (47.4)	678 (50.9)	1225 (45.7)	5.2 (1.9, 8.5)	0.002
Both	17 (0.4)	8 (0.6)	9 (0.3)	0.3 (−0.2, 0.7)	0.22
Feeding route (%)					
Normal diet	1738 (43.3)	–	1738 (64.9)	–	–
Soft diet	942 (23.5)	–	942 (35.1)	–	–
NGT-fed	225 (5.6)	225 (16.9)	–	–	–
PEG-fed	7 (0.2)	7 (0.5)	–	–	–
Intravenous/subcutaneous fluids	726 (18.1)	726 (54.5)	–	–	–
No feeding fluids	373 (9.3)	373 (28.0)	–	–	–
Neuroimaging (%)					
Abnormal scan	3763 (97.6)	1274 (98.9)	2489 (96.9)	2.0 (1.1, 2.9)	<0.001
Location					
Lobar (ACA, MCA, or PCA)	1992 (51.6)	834 (64.8)	1158 (45.1)	19.7 (16.4, 22.9)	<0.001
Lacunar	396 (10.3)	71 (5.5)	325 (12.7)	−7.1 (−8.9, −5.3)	<0.001
Brainstem or cerebellar	73 (1.9)	20 (1.6)	53 (2.1)	−0.5 (−1.4, 0.4)	0.27
Mass effect, moderate to extreme	1178 (31.3)	542 (43.0)	636 (25.4)	17.6 (14.4, 20.8)	<0.001
Previous stroke lesion(s)	2326 (60.5)	763 (59.3)	1563 (61.1)	−1.8 (−5.1, 1.5)	0.28
Cerebral atrophy	3229 (84.0)	1086 (84.4)	2143 (83.8)	0.6 (−1.8, 3.1)	0.63
Leukoaraiosis	1644 (42.8)	566 (44.0)	1078 (42.1)	1.8 (−1.5, 5.2)	0.28

Data are number (%) or mean (standard deviation). Comparison by chi-square test or *t* test.

### Feeding Route and Outcome

**Fig. 2:**
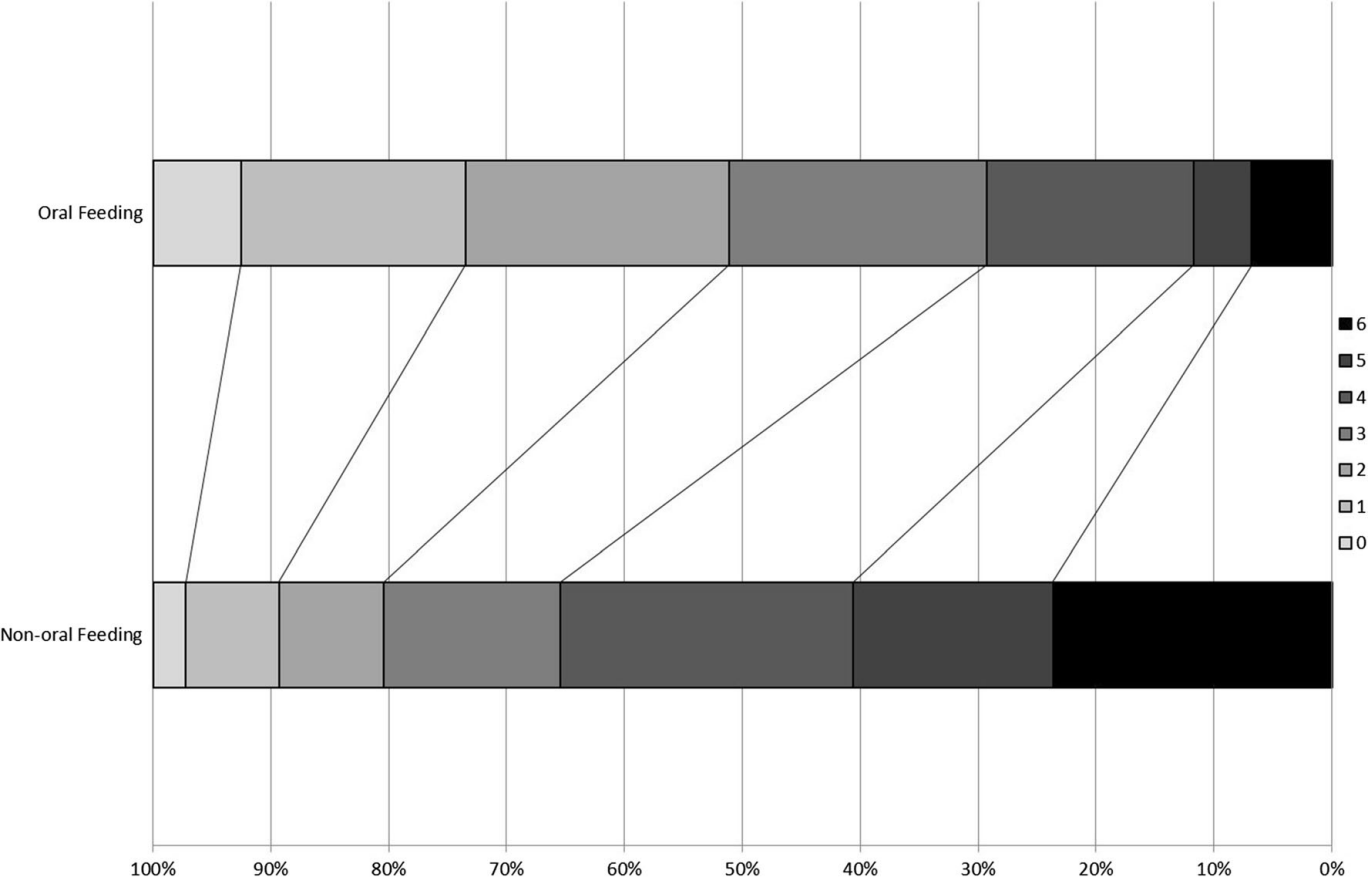
Modified Rankin Scale at day 90. Distribution in modified Rankin Scale at day 90 between patients who were allowed to feed orally versus those who were not allowed to feed orally at baseline. *mRS 0*: independent and no symptoms; *mRS 5*: dependent with full care; *mRS 6*: dead. Comparison by adjusted ordinal logistic regression. Adjusted common odds ratio 1.43 (95% confidence intervals 1.24–1.64, 2*p* < 0.001)

Data on outcomes at days 7 and 90 were missing in 10 (0.25%) and 15 (0.37%) patients, respectively. Non-oral feeding was associated with a worse outcome at day 7 with more deaths (+5.3%), death or neurological deterioration (+11.7%) and worse impairment (SSS −1.3 points) (Table 2). During hospitalisation, patients who were not on oral feeding had a longer hospital stay (+6.8 days; Supplementary Fig. II), and were more likely to develop a respiratory tract infection or pneumonia (+10.9%), be admitted to a stroke rehabilitation unit (+16.7%), be seen by a speech and language therapist (+33.4%), be discharged to an institution (+30.5%) or die in hospital (+14.3%). Similarly, outcomes at day 90 were significantly worse in the non-oral feeding group, manifest as worse dependency (median mRS +1.0 unit, Fig. 2), disability (Barthel Index −7.3 units), cognitive impairment (telephone MMSE −1.0 unit), mood disturbance (ZDS +4.1 unit) and quality of life (health utility status −0.03, EQ-VAS −3.3) (Table 2), as well as a higher rate of death (absolute increase 16.8%; Supplementary Fig. III).

**Table 2:** Comparison of outcomes by feeding route at baseline

	Number	All	No oral feeding	Oral feeding	OR/MD unadjusted	2*p*	OR/MD adjusted	2*p*
Participants			1331	2680				
Day 7								
Death (%)	4001	119 (3.0)	86 (6.5)	33 (1.2)	5.54 (3.69, 8.32)	<0.001	1.64 (1.02, 2.62)	0.040
Death or deterioration (%)	3991	378 (9.5)	229 (17.3)	149 (5.6)	3.52 (2.83, 4.38)	<0.001	1.92 (1.48, 2.48)	<0.001
SSS	3991	38.8 (16.1)	29.0 (17.4)	43.7 (12.9)	−14.7 (−15.6, −13.7)	<0.001	−1.3 (−2.0, −0.5)	<0.001
Hospital								
Length of stay (days)	3985	20.9 (23.6)	30.2 (28.9)	16.3 (18.8)	13.9 (12.4, 15.4)	<0.001	6.8 (5.2, 8.5)	<0.001
RTI, all (%)^a^	4011	257 (6.4)	183 (13.7)	74 (2.8)	5.61 (4.25, 7.42)	<0.001	2.03 (1.46, 2.82)	<0.001
RTI, fatal (%)^a^	4011	149 (3.7)	115 (8.6)	34 (1.3)	7.36 (4.99, 10.86)	<0.001	2.32 (1.48, 3.64)	<0.001
Admitted to SRU (%)	3984	2018 (50.7)	817 (61.8)	1201 (45.1)	1.97 (1.72, 2.26)	<0.001	2.03 (1.73, 2.38)	<0.001
SLT management (%)	3984	1979 (49.7)	951 (72.0)	1028 (38.6)	4.09 (3.54, 4.72)	<0.001	3.51 (2.98, 4.15)	<0.001
To institution (%)	3666	1157 (31.6)	577 (53.0)	580 (22.5)	3.89 (3.35, 4.52)	<0.001	2.37 (1.99, 2.81)	<0.001
Died (%)	4011	320 (8.0)	233 (17.5)	87 (3.2)	6.32 (4.90, 8.17)	<0.001	1.98 (1.47, 2.67)	<0.001
Day 90								
Death (%)	3996	496 (12.4)	314 (23.6)	182 (6.8)	4.23 (3.47, 5.15)	<0.001	1.35 (1.06, 1.71)	0.014
mRS (/6)	3995	3.1 (1.7)	4.0 (1.7)	2.7 (1.6)	1.3 (1.2, 1.4)	<0.001	0.2 (0.1, 0.3)	<0.001
Barthel Index (/100)	3970	64.4 (38.8)	43.1 (40.8)	75.0 (32.9)	−32.0 (−34.3, −29.6)	<0.001	−7.3 (−9.5, −5.0)	<0.001
tMMSE (/19)	2032	11.0 (7.6)	6.8 (8.2)	13.0 (6.4)	−6.2 (−6.8, −5.5)	<0.001	−1.0 (−1.6, −0.3)	0.003
TICS (/37)	2013	14.7 (10.7)	9.1 (11.0)	17.4 (9.3)	−8.4 (−9.3, −7.4)	<0.001	−1.2 (−2.1, −0.3)	0.012
Animal naming	2366	9.3 (7.8)	5.9 (7.5)	10.9 (7.4)	−5.0 (−5.7, −4.4)	<0.001	−0.6 (−1.3, 0.0)	0.068
ZDS	3253	58.5 (24.1)	69.5 (26.5)	54.0 (21.5)	15.5 (13.8, 17.3)	<0.001	4.1 (2.3, 5.9)	<0.001
EQ-5D HUS (/1.0)	3952	0.5 (0.4)	0.3 (0.4)	0.5 (0.4)	−0.3 (−0.3, −0.2)	<0.001	−0.03 (−0.1, 0.0)	0.028
EQ-VAS	3440	56.1 (31.2)	42.5 (34.4)	62.0 (27.7)	−19.5 (−21.6, −17.3)	<0.001	−3.3 (−5.5, −1.1)	0.004
Not at home^b^	3980	1326 (33.3)	751 (56.6)	575 (21.7)	4.72 (4.09, 5.45)	<0.001	2.19 (1.85, 2.59)	<0.001

Data are number (%) or mean (standard deviation) and odds ratio (OR)/mean difference (MD) (95% confidence intervals). Comparison by binary logistic regression, or multiple linear regression, with adjustment

*EQ-5D* Euro-QoL-5 Dimension-3 level, *EQ-VAS* Euro-QoL-Visual Analogue Scale, *HUS* health utility status (derived from EQ-5D), *RTI* respiratory tract infection, *SLT* speech and language therapy, *SRU* stroke rehabilitation unit, *SSS* Scandinavian Stroke Scale, *TICS* telephone interview of cognition scale, *tMMSE* telephone mini-mental state examination, *VAS* visual analogue scale, *ZDS* Zung depression scale

^a^From serious adverse event reports

^b^Dead, still in hospital or in institution

### Feeding Route and Respiratory Tract Infection

RTI was more likely to develop in patients with an altered feeding route, adjusted common odds ratio 1.41, 95% CI 1.12–1.79, 2*p* = 0.004 (Supplementary Fig. IV). In patients with non-oral feeding, RTI was reported in 183 (pneumonia 173, chest infection 9, COPD 1) of 1331 (13.7%) patients, with a median time to presentation of 8.0 [2.0, 29.0] days from entry into the trial (equivalent to ~9 days from stroke onset). Development of pneumonia was not associated with side of stroke: right lesion 127/2091 (6.1%) and left lesion 111/1903 (5.8%) (2*p* = 0.75). Development of RTI was associated with increased discharge to an institution (+29.1%) and death in hospital (+38.6%) (Supplementary Table III). Similarly, outcomes were worse at day 90 with increased death (+54.9%) and worse dependency (mRS +1.0), disability, cognition, mood and quality of life. The negative cognition scores reflect that a majority of patients who were on non-oral feeding and who developed RTI went on to die in hospital.

### Glyceryl Trinitrate and Feeding Route

**Fig. 3:**
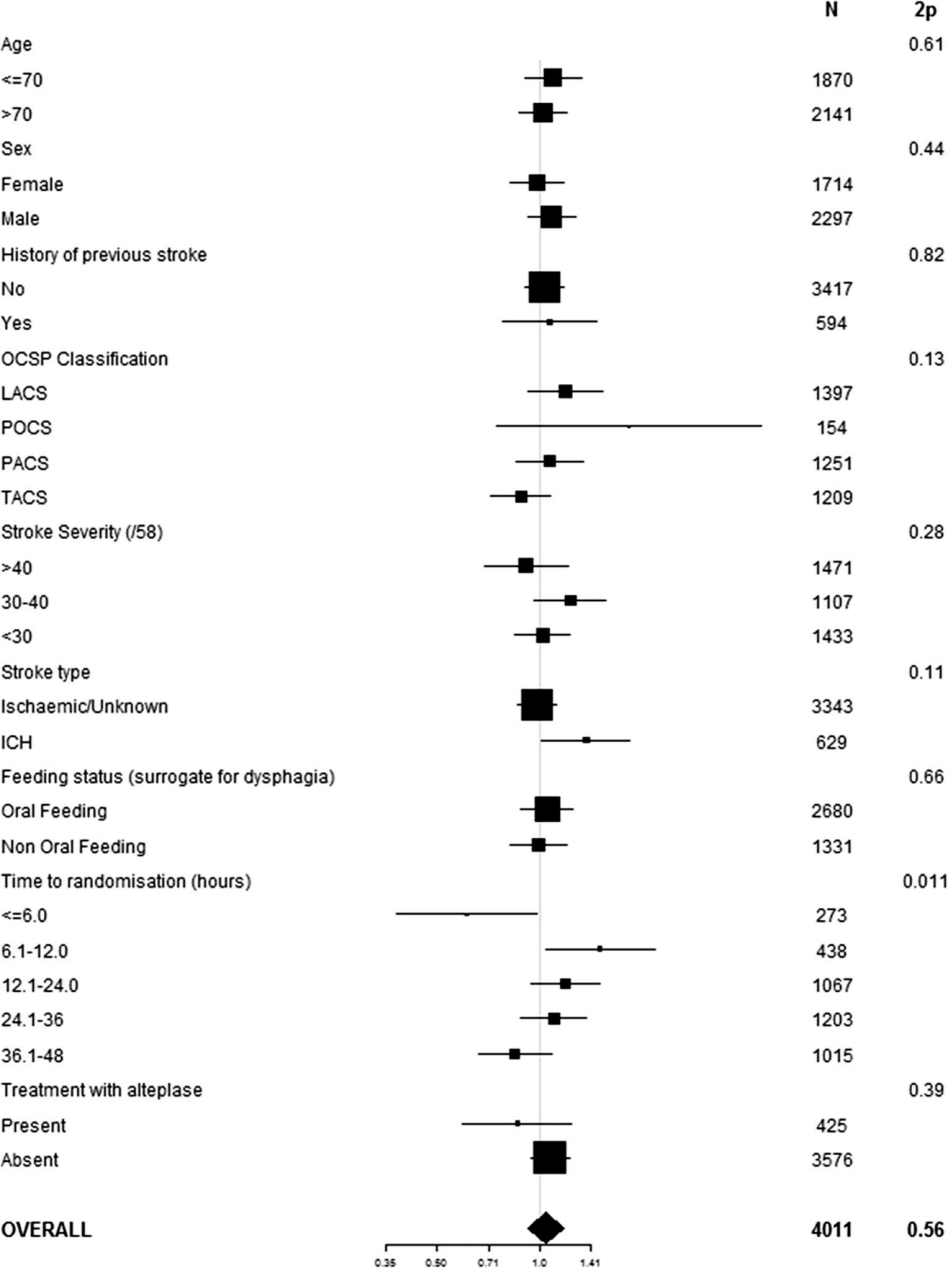
Forest plot of effect of GTN versus no GTN on feeding route in pre-specified subgroups. Comparison with ordinal logistic regression with interaction term added between subgroup and GTN

Overall, GTN had no effect on route of feeding at day 7 whether assessed in unadjusted or adjusted analyses (Supplementary Fig. V). When assessed in pre-specified subgroups [17], an interaction (*p* = 0.011) was present between GTN, feeding route and time to treatment, with GTN appearing to improve feeding route at day 7 in patients randomised within 6 h of stroke onset (Fig. 3). When focussing on this subgroup, randomisation to GTN was associated with a move to improved feeding route, common odds ratio 0.61, 95% confidence intervals 0.38, 0.98; *p* = 0.040 (Table 3; Supplementary Fig. VI) and a tendency to less respiratory tract infection, including pneumonia.

**Table 3:** Comparison of outcomes by randomised treatment, GTN versus no GTN, in patients randomised within 6 h of stroke onset

	Number	All	GTN	No GTN	OR	2*p*
Participants		273	144	129		
Day 7						
Feeding route [/7]^a^	270	1 [1, 2]	1 [1, 2]	1 [1, 2]	0.61 (0.38, 0.98)	0.040
Feeding, non-oral	264	42 (15.9)	18 (12.6)	24 (19.8)	0.58 (0.30, 1.13)	0.11
Hospital						
RTI, all^b^	273	12 (4.4)	3 (2.1)	9 (7.0)	0.28 (0.08, 1.07)	0.063
RTI, fatal	273	8 (2.9)	1 (0.7)	7 (5.4)	0.12 (0.01, 1.00)	0.05
Admitted to SRU	268	101 (37.7)	52 (36.4)	49 (39.2)	0.89 (0.54, 1.45)	0.63
Discharge to institution	243	73 (30.0)	40 (29.6)	33 (30.6)	0.96 (0.55, 1.66)	0.88
Died	273	25 (9.2)	8 (5.6)	17 (13.2)	0.39 (0.16, 0.93)	0.034
Day 90						
Feeding: unable/need help^c^	236	74 (31.4)	36 (27.1)	38 (36.9)	0.63 (0.36, 1.10)	0.11

Data are number (%), median [interquartile range] and odds ratio (OR)/(95% confidence intervals). Comparison by binary logistic regression or ordinal logistic regression

*RTI* respiratory tract infection, *SRU* stroke rehabilitation unit, ^﻿a﻿^Feeding status:*1* normal diet, *2* soft diet, *3* NGT-fed, *4* PEG-fed, *5* IV/SC fluids, *6* no feeding/fluids, *7* death

^b^From serious adverse event reports

^c^From Barthel Index

## Discussion

These pre-specified [17] secondary exploratory analyses of the ENOS trial confirm earlier data that swallowing problems, assessed here using the surrogate of route of feeding, are common immediately after stroke. Feeding route improved over the first week, and was associated with a poor outcome apparent as increased death, dependency, disability, impairment, institutionalisation and pneumonia. Although GTN did not alter feeding route overall, patients randomised to GTN within 6 h of ictus were less likely to require enteral feeding or parenteral fluids.

The observation that post-stroke swallowing problems are common supports earlier studies [4]. Although many patients improved their feeding route over the first week, some did not and a minority worsened, as reported by others [1–3]. Factors associated with increased death and dependency included patient characteristics (increased age, female sex), medical history (more hypertension) and clinical presentation (more cortical syndromes and higher stroke severity and blood pressure), as published previously [4]. Further, dysphagia causes aspiration of foods, liquids and oral secretions and therefore pneumonia [5–7] and death [6], these observations also being seen here.

GTN, if given within 6 h of stroke, improved feeding route at day 7. Although this finding could be due to chance, GTN was associated with improved functional outcome when given hyper-acutely in ENOS [19, 21] and ultra-acutely in the RIGHT pre-hospital trial [22]. This raises the possibility that the improvement in dysphagia (and dependency) results from a reduction in stroke damage due, perhaps, to improved perfusion, blood pressure lowering and/or neuroprotection. In this respect, feeding route is likely to be a biomarker for stroke recovery reflecting a change in phenotype from severe stroke (with swallowing impairment) to a less severe level of stroke and swallowing impairment. Nitric oxide donors also improve exercise tolerance [24] and so might reduce tiring during mastication and swallowing. GTN might also have improved swallowing though relaxing oesophageal smooth muscle thereby improving the oesophageal phase of swallowing, as seen potentially in a small trial of nifedipine [13]. However, such a mechanism would not be expected to be time-dependent and GTN should therefore have improved feeding route irrespective of recruitment time after stroke onset; further, direct effects on smooth muscle would not explain apparent effects seen with less dependency, disability, cognitive impairment and mood disturbance, and improved quality of life [19, 21, 22]. Moreover, in the absence of direct measures of oesophageal physiology such as a barium swallow or manometry, it is not possible to say that oesophageal function was altered by GTN. Indeed, oesophageal relaxation might promote gastro-oesophageal reflux and the development of aspiration pneumonia; in reality, there was no evidence for this across the main trial [19] and a tendency to less pneumonia was seen in those treated early with GTN. As a result, the effect of GTN on feeding route is unlikely to be specific to swallowing.

The strengths of this study are its large size with data coming from a high fidelity trial that recruited more than 4000 patients from 5 continents and 23 countries. Data on feeding route at baseline were complete, and the findings consistent across outcomes. Hence, the results are likely to exhibit both internal and external validity. Nevertheless, several caveats need to be made. First, dysphagia was not routinely diagnosed using techniques such as videofluoroscopy or fibreoptic endoscopy, and clinical decisions on how to feed were based on local guideline-based practice that will have varied considerably across the 173 recruiting sites. As a result, we used route of feeding as a proxy for presumed or diagnosed dysphagia. One common model of care will have consisted of nurses performing a water-based screening test [25]; if swallowing was unsafe, they will have put the patient ‘nil by mouth’, and referred patients on to speech and language therapists for formal swallowing assessment and dieticians for provision of nutrition. Equally, some patients (almost 10% here) received no fluids or feeding over the first week. Although reasons were not collected, some physicians believe that the resulting dehydrating will reduce the development of cerebral oedema in patients with severe stroke; additionally, this may have reflected placing the patient on a care of the dying pathway. Although conceivable in a single-blind trial, it seems unlikely that any of these decisions will have been determined on the basis on randomised ENOS treatments.

Second, the feeding route scale used here is novel and not validated although it was defined prospectively and collected from the start of the trial in 2001 [17]; further, it is easy to assess clinically and its components exhibit face, content and construct validity. A criticism of the use of this scale at baseline is inclusion of PEG-feeding since this is most likely to represent a PEG from before the index stroke as acute PEG insertion within 48 h of stroke onset is not common practice. Third, ENOS mostly included patients with anterior circulation strokes (as is typical for most acute stroke trials) and so those with posterior strokes were underrepresented. Since there are differences in the role of brain regions in controlling swallowing, especially between hemispheres and brain stem, the results seen here may not be representative for posterior circulation strokes. Fourth, although information on the treatment of dysphagia during admission was not collected, more patients with non-oral feeding were seen by a speech and language therapist; this is most likely to have been for swallowing rather than speech problems. Fifth, some patients may have had pre-existing dysphagia, either due to a previous stroke or for another reason, and this information was not collected; 14.8% of patients reported a history of previous stroke and about half of these may have had this complicated by dysphagia, at least during the early phase of recovery. Previous dysphagia would then have made it less likely that patients would recover from the index swallowing problems. Sixth, data on pneumonia and chest infection came from serious adverse event reports and definitions were not operationalised; hence, the number of events will probably have been underestimated. Although investigator reporting of RTI may have been biased by knowledge of treatment assignment, it is unlikely that this would explain the magnitude of effects seen here. Last, information on other dysphagia-related outcomes such as malnutrition, body weight or albumin levels were not collected in the trial.

In summary, the route of feeding was abnormal in a significant minority of patients with acute stroke, with many of these recovering over the first week; non-oral feeding was associated with poor outcome. Trials of potential treatments for post-stroke dysphagia are urgently needed. The potential benefit of GTN on feeding route is being tested as a secondary outcome in the ongoing phase III RIGHT-2 trial of GTN given in the ultra-acute phase after stroke (http://right-2.ac.uk). The route of feeding scale used here is novel and needs further assessment but is pragmatic and uses readily available information so may prove useful in the future.

## Electronic Supplementary Material

ESM 1 (DOCX 227 kb)
