# Supplementary material for: Route of Feeding as a Proxy for Dysphagia After Stroke and the Effect of Transdermal Glyceryl Trinitrate: Data from the Efficacy of Nitric Oxide in Stroke Randomised Controlled Trial
Source: Transl Stroke Res. 2017 Aug 2;9(2):120–9. doi: 10.1007/s12975-017-0548-0 (PMC5849635; doi:10.1007/s12975-017-0548-0)
Supplement: Supplementary file 1 — (DOCX 227 kb) [file 12975_2017_548_MOESM1_ESM.docx]

Route of feeding as a proxy for dysphagia after stroke and the effect of transdermal glyceryl trinitrate: data from the Efficacy of Nitric Oxide in Stroke randomised controlled trial

Lisa J Woodhouse; Polly Scutt; Shaheen Hamdy; David G Smithard; David L Cohen; Christine Roffe; Daniel Bereczki; Eivind Berge; Christopher F Bladin; Valeria Caso; Hanne K Christensen; Rónán Collins; Anna Czlonkowska; Asita de Silva; Anwar Etribi; Ann-Charlotte Laska; George Ntaios; Serefnur Ozturk; Stephen J Phillips; Kameshwar Prasad; Szabolcs Szatmari; Nikola Sprigg; Philip M Bath; for the ENOS Investigators *

METHODS, ADDITIONAL

ENOS trial

The protocol, statistical analysis plan, baseline characteristics, and main results for ENOS (ISRCTN99414122) have been published previously.[^1-4^](#_ENREF_1) Briefly, ENOS examined the safety and efficacy of GTN (5 mg for 7 days) versus no GTN (single-blind delivery) in patients with ischaemic or haemorrhagic stroke, and high blood pressure (systolic BP> 140 mmHg), and with randomisation within 48 hours of onset. Patients who were taking antihypertensive therapy immediately prior to their stroke were also randomised to continue or stop this (open-label, for 7 days) in a partial factorial design. The primary outcome was the modified Rankin Scale (mRS) assessed by masked central telephone call at day 90. The trial recruited 4011 patients from 173 sites across 23 countries in 5 continents.[^3^](#_ENREF_3) Although the trial was neutral for both interventions, patients randomised to GTN within 6 hours, a pre-specified subgroup,[^2^](#_ENREF_2) had a better functional outcome than those assigned to no GTN.[^4^](#_ENREF_4) [^5^](#_ENREF_5)

Statistics

Analyses are shown both unadjusted, and adjusted for age, sex, pre-morbid mRS, Scandinavian Stroke Scale (SSS), total anterior circulation syndrome (TACS), feeding route, and time to randomisation. This list of covariates was determined using a stepwise regression model using mRS as the outcome. Subgroup analyses were performed by adding an interaction term to adjusted models. Since an intervention could reduce dependency but increase death, all ordinal or continuous measures included a score for death, as is standard for mRS, BI and EQ-5D/HUS; death was coded as -5 for BI; -1 for verbal fluency (animal naming), EuroQoL-visual analogue scale (EQ-VAS), telephone mini-mental state examination (t-MMSE), and telephone interview for cognition scale-modified (TICS-M); 0 for health utility status (HUS), as derived from the EuroQoL-5 dimension/3 level (EQ-5D); 6 for mRS; 7 for feeding route; and 102.5 for Zung depression scale (ZDS).[^6^](#_ENREF_6) Ordinal numbers-needed-to-treat (NNTo) were calculated as published [^7^](#_ENREF_7) and signify the number of people that need to be treated for one of them to move by one or more levels of outcome, e.g. from mRS 4 to 3 or lower, or feeding route from nasogastric tube to oral diet.

Supplementary Table I. Baseline characteristics of patients randomised within 6 hours of stroke onset who were on oral versus non oral-feeding/fluids at baseline. Data are number (%) or mean (standard deviation). Comparison by Chi-square test or t-test.

|  | All | Non-oral | Oral | Difference (95% CI) | 2p |
| --- | --- | --- | --- | --- | --- |
| Number of participants | 273 | 117 | 156 |  |  |
| Age (years) | 69.9 (12.7) | 73.8 (12.5) | 66.9 (12.0) | 6.8 (3.9, 9.8) | < 0.001 |
| Sex, male (%) | 154 (56.4) | 57 (48.7) | 97 (62.2) | -13.5 (-25.3, -1.6) | 0.026 |
| Medical history (%) |  |  |  |  |  |
| Previous stroke | 39 (14.3) | 14 (12.0) | 25 (16.0) | -4.1 (-12.3, 4.2) | 0.34 |
| Hypertension | 168 (61.5) | 71 (60.7) | 97 (62.2) | -1.5 (-13.2, 10.2) | 0.80 |
| Ischaemic heart disease | 34 (12.5) | 15 (12.8) | 19 (12.2) | 0.5 (-7.6, 8.6) | 0.90 |
| Scandinavian Stroke Scale | 32.1 (11.9) | 27.9 (11.5) | 35.2 (11.2) | -7.3 (-10.0, -4.6) | < 0.001 |
| Blood pressure (mmHg) |  |  |  |  |  |
| Systolic | 166.7 (18.2) | 165.2 (18.1) | 167.9 (18.2) | -2.7 (-7.0, 1.7) | 0.23 |
| Diastolic | 90.8 (13.3) | 89.5 (13.5) | 91.8 (13.1) | -2.3 (-5.5, 0.9) | 0.17 |
| Heart rate (bpm) | 77.5 (14.9) | 77.9 (15.4) | 77.2 (14.6) | 0.7 (-2.9, 4.3) | 0.69 |
| Stroke type (%) |  |  |  |  |  |
| Ischaemic | 208 (76.2) | 87 (74.4) | 121 (77.6) | -3.2 (-13.5, 7.1) | 0.54 |
| Haemorrhagic | 61 (22.3) | 26 (22.2) | 35 (22.4) | -0.2 (-10.2, 9.8) | 0.97 |
| Time to randomisation (hours) | 4.3 (1.2) | 4.2 (1.2) | 4.4 (1.2) | -0.2 (-0.4, 0.1) | 0.27 |
| Syndrome (%) |  |  |  |  |  |
| Total anterior | 86 (31.5) | 53 (45.3) | 33 (21.2) | 24.1 (13.1, 35.2) | < 0.001 |
| Partial anterior | 112 (41.0) | 47 (40.2) | 65 (41.7) | -1.5 (-13.3, 10.3) | 0.80 |
| Posterior | 13 (4.8) | 4 (3.4) | 9 (5.8) | -2.4 (-7.3, 2.6) | 0.37 |
| Lacunar | 62 (22.7) | 13 (11.1) | 49 (31.4) | -20.3 (-29.5, -11.1) | < 0.001 |
| Symptoms (%) |  |  |  |  |  |
| Dysphasia | 132 (48.4) | 62 (53.0) | 70 (44.9) | 8.1 (-3.8, 20.1) | 0.18 |
| Neglect | 83 (33.1) | 56 (52.8) | 27 (18.6) | 34.2 (22.8, 45.6) | < 0.001 |
| Side of lesion (%) |  |  |  |  |  |
| Right | 150 (54.9) | 59 (50.4) | 91 (58.3) | -7.9 (-19.8, 4.0) | 0.19 |
| Left | 123 (45.1) | 58 (49.6) | 65 (41.7) | 7.9 (-4.0, 19.8) | 0.19 |
| Feeding Route (%) |  |  |  |  |  |
| Normal Diet | 93 (34.1) |  | 93 (59.6) |  |  |
| Soft Diet | 63 (23.1) |  | 63 (40.4) |  |  |
| NGT-fed | 8 (2.9) | 8 (6.8) |  |  |  |
| Intravenous/subcutaneous fluids | 38 (13.9) | 38 (32.5) |  |  |  |
| No feeding fluids | 71 (26.0) | 71 (60.7) |  |  |  |
| Neuroimaging (%) |  |  |  |  |  |
| Abnormal scan | 252 (96.9) | 108 (97.3) | 144 (96.6) | 0.7 (-3.5, 4.8) | 0.76 |
| Location |  |  |  |  |  |
| Lobar (ACA, MCA or PCA) | 122 (46.9) | 55 (49.5) | 67 (45.0) | 4.6 (-7.7, 16.8) | 0.46 |
| Lacunar | 20 (7.7) | 9 (8.1) | 11 (7.4) | 0.7 (-5.9, 7.3) | 0.83 |
| Brainstem or cerebellar | 4 (1.5) | 0 (0.0) | 4 (2.7) | - | - |
| Mass effect, moderate to extreme | 56 (22.1) | 26 (24.3) | 30 (20.5) | 3.8 (-6.7, 14.2) | 0.48 |
| Previous stroke lesion(s) | 163 (62.7) | 73 (65.8) | 90 (60.4) | 5.4 (-6.5, 17.2) | 0.38 |
| Cerebral atrophy | 228 (87.7) | 102 (91.9) | 126 (84.6) | 7.3 (-0.4, 15.0) | 0.075 |
| Leukoaraiosis | 101 (38.8) | 46 (41.4) | 55 (36.9) | 4.5 (-7.5, 16.5) | 0.46 |

Supplementary Table II. Change in feeding route from baseline to day 7. Data are number (%).

| Baseline | Oral |  |  |  | Non-oral |  |
| --- | --- | --- | --- | --- | --- | --- |
|  | Normal  diet | Soft  diet | NGT-fed | PEG-fed | Iv/sc  fluids | No feed  / fluids |
| *Day 7* |  |  |  |  |  |  |
| Normal diet | 1577 (90.7) | 434 (46.1) | 38 (16.9) | 0 (0.0) | 167 (23.0) | 135 (36.2) |
| Soft diet | 114 (6.6) | 425 (45.1) | 52 (23.1) | 2 (28.6) | 250 (34.4) | 112 (30.0) |
| NGT-fed | 22 (1.3) | 41 (4.4) | 110 (48.9) | 3 (42.9) | 187 (25.8) | 68 (18.2) |
| PEG-fed | 2 (0.1) | 0 (0.0) | 2 (0.9) | 1 (14.3) | 3 (0.4) | 4 (1.1) |
| Iv/sc fluids | 4 (0.2) | 10 (1.1) | 2 (0.9) | 1 (14.3) | 58 (8.0) | 22 (5.9) |
| No feed  / fluids | 2 (0.1) | 5 (0.5) | 4 (1.8) | 0 (0.0) | 15 (2.1) | 6 (1.6) |
| Death | 11 (0.6) | 22 (2.3) | 16 (7.1) | 0 (0.0) | 44 (6.1) | 26 (7.0) |
| Missing | 6 (0.3) | 5 (0.5) | 1 (0.4) | 0 (0.0) | 2 (0.3) | 0 (0.0) |

Supplementary Table III. Comparison of outcomes in patients with non-oral feeding at baseline who developed or did not develop respiratory tract infection (RTI), according to serious adverse event reporting. Data are number (%) or mean (standard deviation), and odds ratio/mean difference (MD) (95% confidence intervals). Comparison by adjusted binary logistic regression, or multiple linear regression.

|  | N | All | RTI | No RTI | OR/MD  unadjusted | 2p | OR/MD  adjusted | 2p |
| --- | --- | --- | --- | --- | --- | --- | --- | --- |
| Participants |  | 1331 | 183 | 1148 |  |  |  |  |
| *Hospital* |  |  |  |  |  |  |  |  |
| Length of stay | 1321 | 30.2 (28.9) | 31.8 (27.8) | 29.9 (29.1) | 1.9 (-2.7, 6.4) | 0.42 | -4.3 (-8.9, 0.4) | 0.071 |
| Discharge to institution | 1088 | 577 (53) | 71 (79.8) | 506 (50.7) | 3.84 (2.26, 6.54) | < 0.001 | 2.58 (1.48, 4.48) | < 0.001 |
| Died | 1331 | 233 (17.5) | 93 (50.8) | 140 (12.2) | 7.44 (5.3, 10.45) | < 0.001 | 4.73 (3.24, 6.92) | < 0.001 |
| *Day 90* |  |  |  |  |  |  |  |  |
| Death (%) | 1328 | 314 (23.6) | 130 (71) | 184 (16.1) | 12.81 (8.97, 18.29) | < 0.001 | 8.32 (5.62, 12.31) | < 0.001 |
| mRS (/6) | 1328 | 4 (1.7) | 5.5 (1) | 3.7 (1.6) | 1.8 (1.5, 2) | < 0.001 | 1.0 (0.8, 1.3) | < 0.001 |
| Barthel Index (/100) | 1324 | 43.1 (40.8) | 4.2 (21.1) | 49.3 (39.7) | -45.1 (-51, -39.2) | < 0.001 | -25.7 (-30.8, -20.7) | < 0.001 |
| tMMSE (/19) | 654 | 6.8 (8.2) | -0.3 (3) | 8.8 (8.1) | -9.1 (-10.5, -7.7) | < 0.001 | -4.7 (-6, -3.5) | < 0.001 |
| TICS (/37) | 648 | 9.1 (11) | -0.1 (4.2) | 11.5 (11) | -11.6 (-13.5, -9.8) | < 0.001 | -5.8 (-7.5, -4.1) | < 0.001 |
| Animal naming | 767 | 5.9 (7.5) | 0 (3.4) | 7.2 (7.6) | -7.3 (-8.6, -6) | < 0.001 | -3.5 (-4.7, -2.4) | < 0.001 |
| ZDS | 963 | 69.5 (26.5) | 97.7 (14.3) | 64.4 (24.9) | 33.3 (29.1, 37.4) | < 0.001 | 22.6 (18.5, 26.6) | < 0.001 |
| EQ-5D HUS (/1.0) | 1311 | 0.3 (0.4) | 0 (0.2) | 0.3 (0.4) | -0.3 (-0.4, -0.2) | < 0.001 | -0.1 (-0.2, -0.1) | < 0.001 |
| EQ-VAS | 1044 | 42.5 (34.4) | 7.7 (21.6) | 48.7 (32.5) | -40.9 (-46.2, -35.7) | < 0.001 | -26.6 (-31.6, -21.5) | < 0.001 |
| Not at home ‡ | 1326 | 751 (56.6) | 167 (91.3) | 584 (51.1) | 9.99 (5.9, 16.9) | < 0.001 | 5.81 (3.35, 10.09) | < 0.001 |

EQ-5D: Euro-QoL-5 Dimensions-3 level; EQ-VAS: Euro-QoL-Visual Analogue Scale; HUS: health utility status (derived from EQ-5D); SSS: Scandinavian Stroke Scale; TICS: telephone interview of cognition scale; tMMSE: telephone mini-mental state examination; VAS: visual analogue scale; ZDS: Zung depression scale.

† From serious adverse event reports. ‡ Died, still in hospital, or in institution

Supplementary Figure I. CONSORT flow diagram for patients with non-oral and oral feeding.

Supplementary Figure II. Length of stay in hospital by feeding route at baseline. Comparison by Spearman’s rank correlation. Spearman’s ρ=0.258, 2p=<0.001.

Supplementary Figure III. Survival plot between patients who were allowed to feed orally versus those who were not allowed to feed orally at baseline. Comparisons by adjusted Cox proportional hazard regression, adjusted hazard ratio 1.33 (95% confidence intervals 1.07-1.64, 2p=0.010).


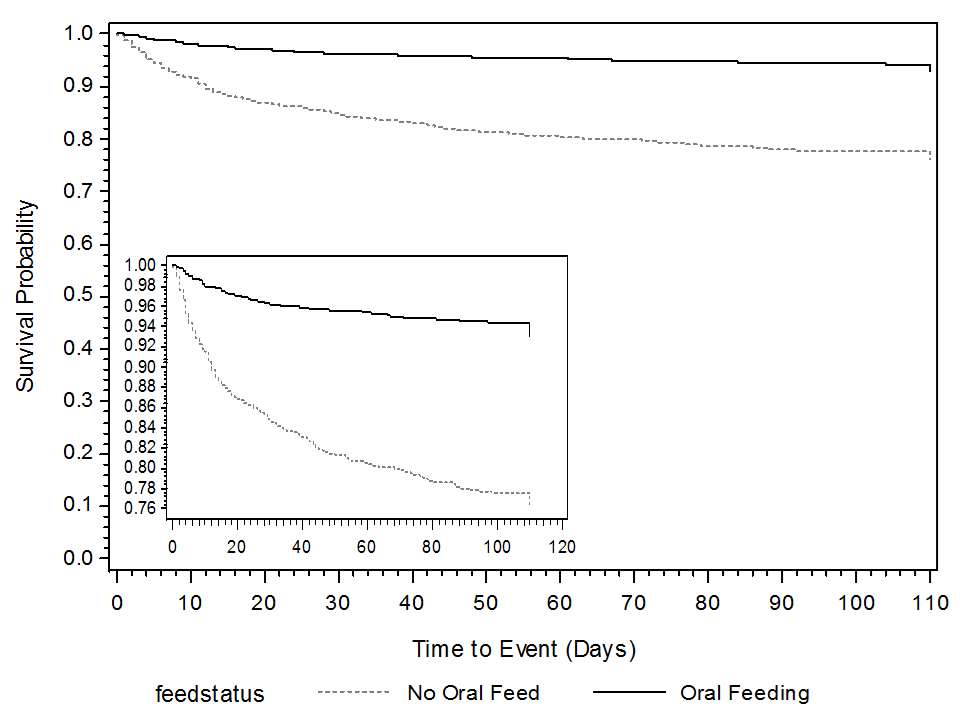


Supplementary Figure IV. Distribution of feeding route at baseline in patients who did, or did not, subsequently develop RTI within 90 days. Comparison by adjusted ordinal logistic regression. Adjusted common odds ratio 1.41 (95% CI 1.12-1.79, 2p=0.004).

Supplementary Figure V. Effect of GTN vs no GTN on shift in feeding route at day 7 for all participants randomised into ENOS. Scale: 1 – normal diet, 2 – soft diet, 3 – NGT fed, 4 – PEG-fed, 5 – iv/sc fed, 6 – no feeding, 7 – died. Comparison by adjusted ordinal logistic regression, adjusted common odds ratio 1.08 (95% confidence intervals 0.94-1.24, 2p=0.34).

Supplementary Figure VI. Effect of GTN vs no GTN on distribution of feeding route at day 7 for participants randomised within 6 hours of stroke onset. Scale: 1 – normal diet, 2 – soft diet, 3 – NGT fed, 4 – PEG-fed, 5 – iv/sc fed, 6 – no feeding, 7 – died. Comparison by ordinal logistic regression. Note, the first 40% of patients were all on normal diet and are not shown for clarity. Common odds ratio 0.61 (95% confidence intervals 0.38, 0.98; p=0.040). Ordinal number needed to treat 7.8 (95% confidence intervals 3.9-2278.8).

REFERENCES

1. The ENOS Trial Investigators. Glyceryl trinitrate vs. control, and continuing vs. stopping temporarily prior antihypertensive therapy, in acute stroke: rationale and design of the Efficacy of Nitric Oxide in Stroke (ENOS) trial (ISRCTN99414122). *International Journal of Stroke* 2006;1:245-49.

2. Bath P, Houlton A, Woodhouse L, et al. Statistical analysis plan for the ‘Efficacy of Nitric Oxide in Stroke’ (ENOS) trial. *International Journal of Stroke* 2014;9(3):372-4. doi: 10.1111/ijs.12235

3. ENOS Investigators. Baseline Characteristics of the 4011 patients recruited into the "efficacy of Nitric Oxide in Stroke (ENOS) trial. *International Journal of Stroke* 2014;9(6):711-20. doi: 10.1111/ijs.12308 [published Online First: 9th July 2014]

4. Bath P, Woodhouse L, Scutt P, et al. Efficacy of nitric oxide, with or without continuing antihypertensive treatment, for management of high blood pressure in acute stroke (ENOS): a partial-factorial randomised controlled trial. *The Lancet* 2015;385(9968):617-28. doi: <http://dx.doi.org/10.1016/S0140-6736(14)61121-1> [published Online First: 22 October 2014]

5. Woodhouse L, Scutt P, Krishnan K, et al. Effect of hyperacute administration (within 6 hours) of transdermal glyceryl trinitrate, a nitric oxide donor, on outcome after stroke: subgroup analysis of the Efficacy of Nitric Oxide in Stroke (ENOS) trial. *Stroke* 2015;46:3194-201.

6. Ankolekar S, Renton C, Sare G, et al. Relationship between Poststroke Cognition, Baseline Factors, and Functional Outcome: Data from "Efficacy of Nitric Oxide in Stroke" Trial. *Journal of Stroke and Cerebrovascular Diseases* 2014;23(7):1821-9. doi: 10.1016/j.jstrokecerebrovasdis.2014.04.022 [published Online First: 21 June 2014]

7. Optimising the Analysis of Stroke Trials (OAST) Collaboration. Calculation of Numbers-Needed-to-Treat (NNT) in parallel group trials assessing ordinal outcomes: Case examples from acute stroke and stroke prevention *International Journal of Stroke* 2011;6(6):472-9.
